# Identifying the Relationship between PM_2.5_ and Hyperlipidemia Using Mendelian Randomization, RNA-seq Data and Model Mice Subjected to Air Pollution

**DOI:** 10.3390/toxics11100823

**Published:** 2023-09-29

**Authors:** Yixue Zhao, Geng Shen, Xipeng Lin, Long Zhang, Fangfang Fan, Yan Zhang, Jianping Li

**Affiliations:** 1 Division of Cardiology, Peking University First Hospital, Beijing 100034, China; 2211110277@pku.edu.cn (Y.Z.); shengeng50@163.com (G.S.); lxp101@pku.edu.cn (X.L.); zhanglongbill@163.com (L.Z.); fang9020@126.com (F.F.); drzhy1108@vip.163.com (Y.Z.); 2 Institute of Cardiovascular Disease, Peking University First Hospital, Beijing 100034, China; 3 State Key Laboratory of Vascular Homeostasis and Remodeling, Beijing 100191, China

**Keywords:** PM_2.5_, air pollution, hyperlipidemia, familial hypercholesterolemia, cardiovascular disease, mendelian randomization, RNA-seq, model mice

## Abstract

Air pollution is an important public health problem that endangers human health. However, the casual association and pathogenesis between particles < 2.5 μm (PM_2.5_) and hyperlipidemia remains incompletely unknown. Mendelian randomization (MR) and transcriptomic data analysis were performed, and an air pollution model using mice was constructed to investigate the association between PM_2.5_ and hyperlipidemia. MR analysis demonstrated that PM_2.5_ is associated with hyperlipidemia and the triglyceride (TG) level in the European population (IVW method of hyperlipidemia: OR: 1.0063, 95%CI: 1.0010–1.0118, *p* = 0.0210; IVW method of TG level: OR: 1.1004, 95%CI: 1.0067–1.2028, *p* = 0.0350). Mest, Adipoq, Ccl2, and Pcsk9 emerged in the differentially expressed genes of the liver and plasma of PM_2.5_ model mice, which might mediate atherosclerosis accelerated by PM_2.5_. The studied animal model shows that the Paigen Diet (PD)-fed male LDLR^−/−^ mice had higher total cholesterol (TC), TG, and CM/VLDL cholesterol levels than the control group did after 10 times 5 mg/kg PM_2.5_ intranasal instillation once every three days. Our study revealed that PM_2.5_ had causality with hyperlipidemia, and PM_2.5_ might affect liver secretion, which could further regulate atherosclerosis. The lipid profile of PD-fed Familial Hypercholesterolemia (FH) model mice is more likely to be jeopardized by PM_2.5_ exposure.

## 1. Introduction

Air pollution is an important public health problem that jeopardizes human health. Particles < 2.5 μm (PM_2.5_) come from the combustion of coal, oil, gasoline, and transformation products of nitrogen oxides (NOx), sulfur dioxide (SO_2_) [1], and are composed of sulfate, nitrate, ammonium, hydrogen ion, elemental carbon, organic compounds, polycyclicaromatichydrocarbons (PAH), metals, particle-bound water, and biogenic organic [2]. PM_2.5_ can be deposited in the respiratory bronchioles and alveoli, in which gas exchange occurs [3]. These particles can affect gas exchange, penetrate the lungs, and escape into the bloodstream, causing significant cardiovascular problems [4].

Arteriosclerotic cardiovascular disease (ASCVD) is the leading cause of human mortality around the world [5], and dyslipidemia composed of hypercholesterolemia, hypertriglyceridemia, hypoalphalipoproteinemia, and hyperbetalipoproteinemia is the major cause of atherosclerosis [6]. Numerous studies have shown that air pollution could affect lipid metabolism and cardiovascular disease incidence and mortality; however, the results are inconsistent [7,8,9,10,11,12,13,14,15,16,17,18,19,20,21,22,23,24,25,26,27,28] (Table S1). Yang et al. conducted a large-scale epidemiological study of 15,477 subjects in 33 communities in China and found that long-term ambient air pollution is associated with dyslipidemia, especially among overweight or obese patients [25]. PM_2.5_ is positively associated with total cholesterol (TC), triglyceride (TG), and low-density lipoprotein cholesterol (LDL-C) and negatively associated with high-density lipoprotein-cholesterol (HDL-C) in the study by Zhang et al. [23]. Nevertheless, Mao et al. demonstrated that the increment of PM_2.5_ is related to the increase in TC, LDL-C, hypercholesterolemia, hyperbetalipoproteinemia, and hypoalphalipoproteinemia, and associated with the decrease in TG and HDL-C [27]. Therefore, the relationship between exposure to atmospheric fine particulate matter and plasma TC is mostly positive, while the relationship between exposure and TG is positive in some studies [23,25,28] and negative in others [19,27,29]. Regarding the air pollution animal model, Song et al. found that after exposure to PM_2.5_ and filtered air (FA), TG and TC levels were increased, and HDL was decreased in both C57BL/6 and db/db mice [30]. The average concentrations of PM_2.5_ in the exposure chamber and FA chamber were 324.2 ± 45.2 μg/m^3^ and 17.3 ± 3.7 μg/m^3^ [30].

Meanwhile, there is still a lack of studies on PM_2.5_ and hyperlipidemia at the human genome-wide level. Transcriptome sequencing analysis in animal model studies and the distribution of lipids and lipoproteins in model animals are seldom reported. We attempted to analyze the association between PM_2.5_-related gene loci and hyperlipidemia gene loci through the GWAS database and explore the association between PM_2.5_ exposure and hyperlipidemia through transcriptome data and laboratory test results via air pollution model mice.

## 2. Materials and Methods

### 2.1. Data Sources

PM_2.5_-related genetic instruments were extracted from a large GWAS study with 423,796 samples consisting of 9,851,867 SNPs of European people (GWAS trait ID: ukb-b-10817). The same hyperlipidemia and total triglycerides genetic instruments from other GWAS studies were used (GWAS trait ID: ukb-b-17462 and met-d-Total_TG).

The RNA-sequencing data of mice liver and plasma were acquired from the Gene Expression Omnibus database (GEO database: GSE146508).

### 2.2. Mendelian Randomization Analysis

We chose SNPs as internal instrumental variables at the *p* < 1 × 10^−5^ significance level, which showed a low possibility of weak instrumental variable bias in MR analysis since there were only 8 SNPs screened at the genome-wide significant threshold of 5 × 10^−8^ [31].

MR methods, including simple median, weighted median (WM), inverse variance weighted (IVW), MR-Egger, weighted mode, and simple mode, were selected to evaluate the causal effect between PM2.5 and hyperlipidemia. Among them, IVW is the major analysis method [32]. The weighted median method [33] and MR Egger [32] methods were conducted for sensitivity analysis to account for potential bias from unknown pleiotropy. The MR Egger estimate is less precise than that from IVW because the variance of the MR Egger estimate is additionally affected by the variability between the genetic associations with exposure or gene polymorphism.

A heterogeneity test was conducted using Cochran’s Q-test to identify whether the MR results were biased by potential heterogenic factors. A leave-one-out permutation test was performed to assess whether the IVW estimate was biased by the influence of particular SNPs. Causal estimates between PM_2.5_ and the hyperlipidemia risk were expressed as odds ratios (OR) and a 95% confidence interval (CI) per standard deviation increment. All the analyses with *p* < 0.05 were considered statistically significant. All statistical analyses were performed using the R Studio (R version 4.3.0) software and the R package “TwoSampleMR”.

### 2.3. Enrichment Analysis

To further investigate the biological mechanisms of DEGs, GO, KEGG, and GSEA analysis was conducted using the “ClusterProfiler” R package. The three categories assessed via GO analyses were as follows: biological process (BP), cellular component (CC), and molecular function (MF), which demonstrated the molecular biological function of the selected genes. The STRING APP of Cytoscape v.3.8.2 was used to conduct the PPI analysis of intersecting DEGs.

### 2.4. Estimation of Immune Cells Infiltration in Mice Livers

CIBERSORT tools were used to explore the difference in immune cell marker expression in mice liver between the cases and controls. The NCBI reference set for 22 immune cell subtypes CIBERSORT regarding gene expression features was used.

### 2.5. Preparation of PM2.5

The PM2.5 sample was bought from the National Institute of Standards and Technology (NIST.SRM 1649b, Urban Dust, USA, http://www.nist.gov/srm accessed on 1 January 2022); PM2.5 was diluted with 0.9% normal saline. The final concentration of PM_2.5_ was 2 mg/mL [34].

### 2.6. Animal Model

Ten 7-week-old male C57BL/6J mice weighing 20–25 g were purchased from Beijing HFK Bio-Technology Company, and twelve 7-week-old male LDLR^−/−^ mice weighing 20–25 g were obtained from State Key Laboratory of Vascular Homeostasis and Remodeling (Peking University, Beijing, China). The sample size was calculated according to the sample size formula [35]. All animals were allowed to adapt to the animal room environment for one week before the study. C57BL/6J mice were fed a chow diet during the air pollution model construction. Eight-week-old LDLR^−/−^ mice were fed a Paigen diet (PD, RESEARCH DIETS, D12109C, New Brunswick, NJ, USA; HF Rodent Diet with Regular Casein, 1.25% Added Cholesterol and 0.5% Sodium Cholate) for 6 weeks to further assess the effect of PM_2.5_ on the plasma lipids. Mice of each strain were randomly assigned to two groups via a randomized block design: PM_2.5_ and saline group.

Intratracheal installation is a more convenient method and can be used to easily calculate exposure doses than exposure chambers can in an air pollution study [36,37]. According to the lung surface area–dose exchange algorithm [34,38], we converted the human PM_2.5_ exposure dose (500 μg/m^3^) into the corresponding mouse dose (5 mg/kg). The intratracheal instillation was performed on 8-week-old C57BL/6J mice and 8-week-old LDLR^−/−^ mice after two weeks of being fed a chow diet (CD) or PD, respectively. During the procedure, the mice were anesthetized with sodium pentobarbital (50 mg/kg) via intraperitoneal injection, and a rodent respirator (ALCV9A; Shanghai Alcott Biotech Co., Ltd., Shanghai, China) was used for ventilation test to ensure successful tracheal intubation. The mice in the PM_2.5_ group were given 5 mg/kg PM_2.5_ [39,40,41] (in 50 μL 0.9% normal saline) once every three days, 10 times [42]; mice in the control group were given 50 μL 0.9% normal saline at the same frequency. CD and PD feeding continued during the instillation period. After 6 weeks of eating a CD or PD, the mice were anesthetized with 1% pentobarbital sodium, the plasma and organs were collected for further analysis, and all mice were sacrificed because of the excessive loss of blood. All procedures were followed to the guidelines of Laboratory Animal Care (NIH Publication No. 85Y23, revised 1996), and the experimental protocol was approved by the Animal Care Committee, Peking University First Hospital (J2022109).

### 2.7. The Assays of Plasma Lipids and Lipoproteins

Blood samples were collected from the retro-orbital plexus of the mice after 4 h fasting under sodium pentobarbital anesthesia. The plasma TC and TG levels were enzymatically determined using commercially available kits (100000180, Total cholesterol Assay Kit (CHOD-PAP), 100000220, calibration product 150 mg/Dl–230 mg/dL; Triglyceride Assay Kit (TG) Enzyme colorimetry (GPO-PAP), calibration product, 1.2 mmol/L–3.2 mmol/L, Zhongsheng Beikong, Beijing, China).

To analyze the lipid distribution, the fast protein liquid chromatography (FPLC) of plasma lipoproteins was performed using 200 μL of pooled plasma samples from the animals of each group with indicated genotypes, which were filtered using 0.22 mm filters and then applied to Tricorn high-performance Superose S-6 10/300 GL columns (Amersham Biosciences), eluting with PBS at a constant flow rate of 0.25 mL/min. The eluted fractions (500 μL per fraction) were assessed for TG and cholesterol concentrations using the same TG and cholesterol kits described above.

### 2.8. qPCR

The total RNA of the mice livers was isolated using TRIzol Reagent (ET111-01, TransGen Biotech, Beijing, China). Briefly, the total RNA was reverse-transcribed to cDNA using the Reverse Transcription Reagent Kit (AH301-02, TransGen Biotech, Beijing, China). The resulting cDNA was amplified via 40 cycles of qPCR using Top Green qPCR SuperMix (AQ132-24, TransGen Biotech, Beijing, China). The mRNA level of each target gene was normalized to an endogenous β-actin expression. The ΔΔCt method was used to evaluate relative expression levels or fold changes. The primer sequences used in our study are listed in Table S2.

### 2.9. Statistical Analysis

All data in the animal model passed the normality test (Shapiro–Wilk test and Kolmogorov–Smirnov test) and are presented as the means ± SEM. Statistical comparisons were performed using an unpaired two-tailed *t*-test. GraphPad Prism 9.0 software was used for statistical analyses. A value of *p* < 0.05 indicates a statistically significant difference.

## 3. Results

### 3.1. Mendelian Randomization Indicates Genetic Association with PM_2.5_ and Hyperlipidemia

In general, in this MR study, we analyzed a total of 423,796 European individuals. We extracted IVs that were significantly associated with glutamine from the GWAS (*p* < 1 × 10^−5^).

**Figure 1:**
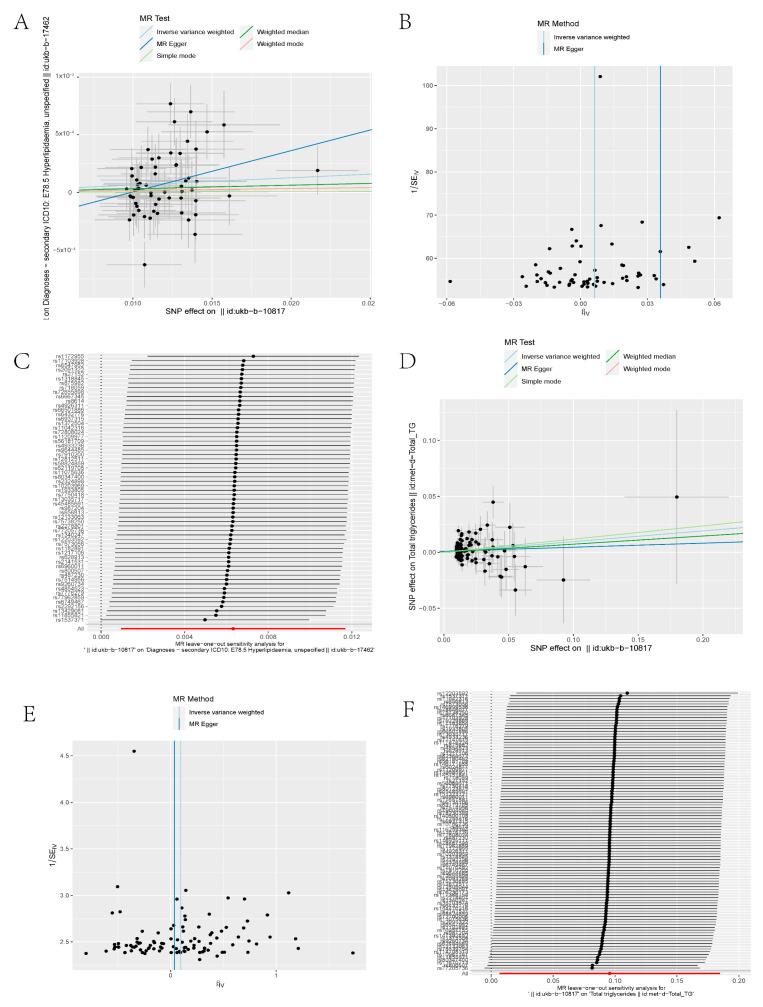
(**A**) Scatter plot of SNPs related to PM_2.5_ and hyperlipidemia. The slope of each line demonstrates the estimated effect of the Mendelian randomization method. (**B**) Funnel plot of PM_2.5_ and hyperlipidemia. Vertical lines represent estimates with all SNPs. Symmetry of the IVW method in the funnel plot demonstrates no obvious horizontal pleiotropy. (**C**) Leave-one-out analysis of PM_2.5_ and hyperlipidemia. There was no substantial change in the IVW causal estimate after removing any of the instrumental SNPs. (**D**) Scatter plot of SNPs related to PM_2.5_ and TG level. (**E**) Funnel plot of PM_2.5_ and TG level. (**F**) Leave-one-out analysis of PM_2.5_ and TG levels. SNP, single-nucleotide polymorphism; IVW, inverse variance weighted; TG, triglyceride.

As shown in Table 1 and Figure 1A, the MR analyses revealed causal associations between PM_2.5_ and hyperlipidemia in the European cohort. The casual inference of genetic liability between PM_2.5_ and hyperlipidemia in the European population was noted (Table 1 and Figure 1A). The casual inference of genetic liability between PM_2.5_ and the TG level in the European population was noted as well (Table 2 and Figure 1D).

Sensitivity analyses for MR were performed. Using leave-one-out analysis, we discovered no single SNP that drove the causal link between PM_2.5_ and hyperlipidemia/the TG level (Figure 1C,F). We performed a pleiotropy test to investigate horizontal pleiotropy (Figure 1B,E), and the results confirmed that pleiotropy was unlikely to bias the causal relationship (*p* > 0.05).

Moreover, bidirectional Mendelian randomization was conducted to analyze the reverse causal relationships between PM_2.5_ and hyperlipidemia/the TG level. Although Mendelian randomization analysis pointing to PM_2.5_ can cause hyperlipidemia or affect the TG level, hyperlipidemia or the TG level does not lead to a high risk of PM_2.5_ exposure.

The significant SNPs of PM_2.5_ were mapped to 34 genes using NCBI. Then, we evaluated the DEGs in the liver of air pollution model mice from GSE146508 of the GEO database and found that Clcn1 was differentially expressed in PM_2.5_ and the control groups.

### 3.2. RNA-seq Data of Liver and Plasma in Air Pollution Model Mice Revealed Hub Genes in Muscle Contraction and Lipid Metabolism

**Figure 2:**
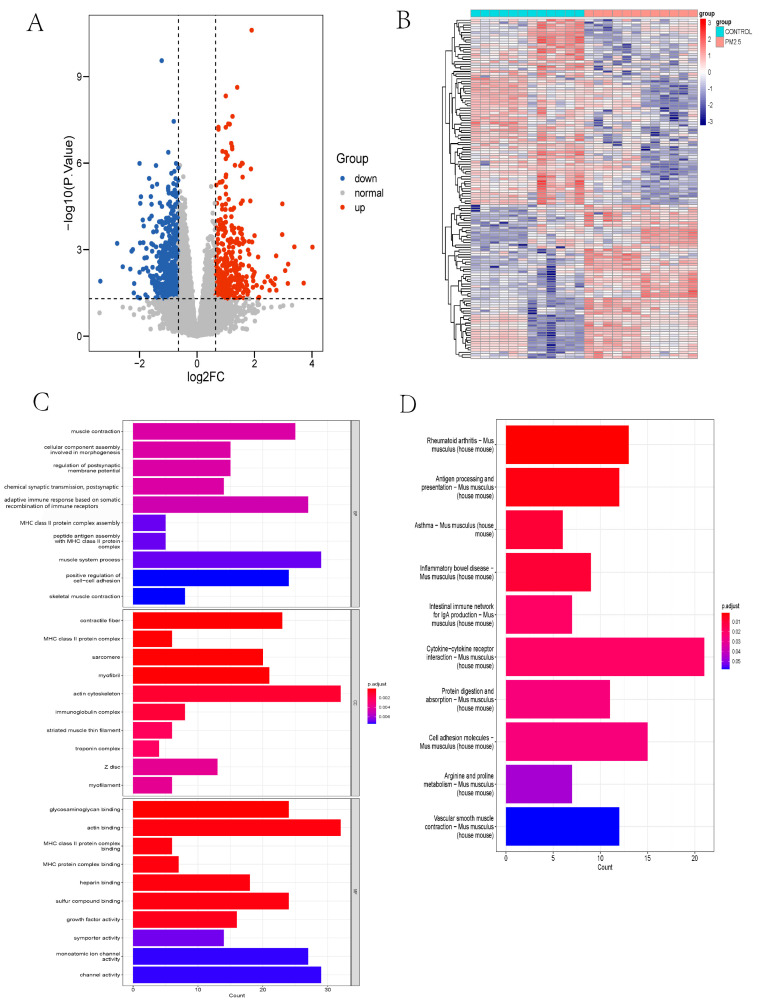
(**A**) Volcano plot of DEGs in PM_2.5_-exposed mice liver and controls from GSE146508. The cutoff for logFC is 0.645. The number of the up gene is 706, and the number of the down gene is 515. (**B**) Heatmap of DEGs in PM_2.5_ exposed mice liver and controls from GSE146508. (**C**) Top 10 pathways of GO enrichment analysis (BP, CC, and MF) about DEGs in PM_2.5_ exposed mice liver and controls from GSE146508. (**D**) Top 10 pathways of KEGG enrichment analysis about DEGs in PM_2.5_-exposed mice liver and controls from GSE146508. DEG, differentially expressed gene; GO, Gene Ontology; BP, biological process; CC, cellular component; and MF, molecular function; KEGG, Kyoto Encyclopedia of Genes and Genomes.

The “limma” package of R software was utilized to analyze the differential expressed genes (DEGs) in the liver and plasma RNA samples from mice of the GSE146508 set. The cutoff for log2FC in the liver is 0.645; 706 genes were downregulated, and 515 genes were upregulated (Figure 2A). The DEGs of the liver are displayed in the heatmap (Figure 2B).

**Figure 3:**
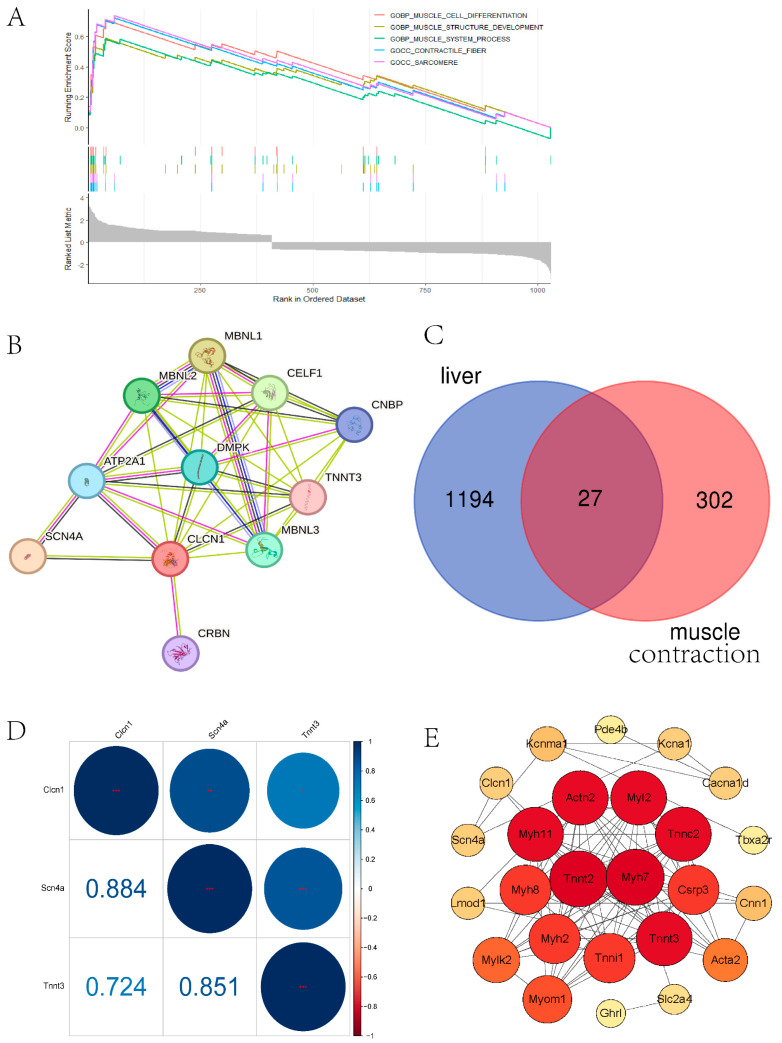
(**A**) Protein–protein interaction network (PPI) of Clcn1 in humans. (**B**) Venn plot of DEGs in PM_2.5_-exposed mice liver and muscle contraction related genes. (**C**) Top 5 pathways of GSEA enrichment analysis about DEGs in PM_2.5_-exposed mice liver and controls from GSE146508. (**D**) Correlation between hub genes Clcn1, Scn5a, and Tnnt3, which all exist in humans and mice. The correlation coefficients are shown in the square. (**E**) PPI network of muscle contraction-related genes arranged by degree using Cytoscape. A large, red circle means that the gene is more important.

To reveal the interaction between the DEGs in the livers of model mice exposed to air pollution, we conducted GO, KEGG, and GSEA enrichment and PPI network analysis. In the GO-BP analysis (Figure 2C), the recombination of immune receptors built from the immunoglobulin superfamily, muscle contraction, and muscle system processes were most significantly enriched. In the GO-CC analysis (Figure 2C), the major pathways were associated with the actin cytoskeleton, contractile fiber, and myofibril. The results of GO-MF (Figure 2C) mainly refer to actin binding, compound binding, and channel activity. These results of the GO enrichment analysis revealed that muscle contraction might play a prominent role in PM_2.5_-exposed mice, in which Clcn1 took part. Meanwhile, KEGG analysis enriched the pathway of cytokine-cytokine receptor interaction and vascular smooth muscle contraction (Figure 2D). GSEA denoted a similar result as GO and KEGG analysis (Figure 3A).

Clcn1-related genes in humans were mapped using STRING: functional protein association networks (string-db.org) (Figure 3B)

We overlapped the mice liver DEGs and muscle contraction-related genes from NCBI (Figure 3C) and illustrated the PPI network using Cytoscape tools (Figure 3E). A correlation test was performed for the hub genes Clcn1, Scn4a, and Tnn3, which all exist in human and mouse data (Figure 3D). 

**Figure 4:**
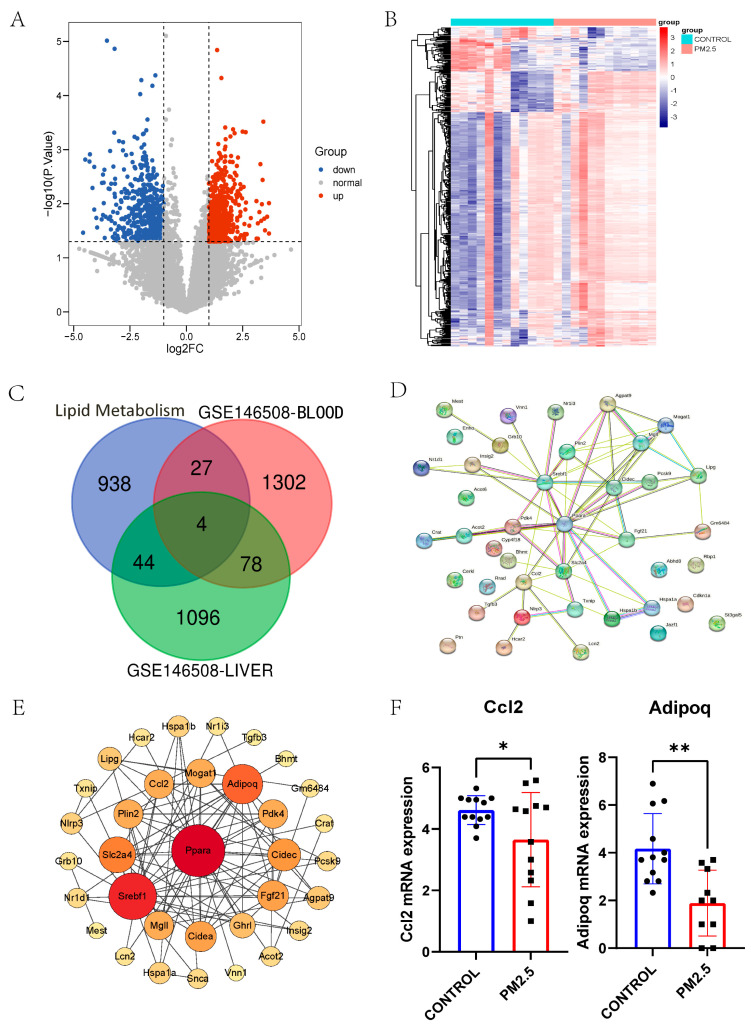
(**A**) Volcano plot of DEGs in PM_2.5_-exposed mice plasma and controls from GSE146508. The cutoff for logFC is 1.000. The number of the up gene is 858, and the number of the down gene is 552. (**B**) Heatmap of DEGs in PM_2.5_-exposed mice plasma and controls from GSE146508. (**C**) Venn plot of DEGs in PM_2.5_-exposed mice liver, plasma, and lipid metabolism-related genes. (**D**) PPI network of lipid metabolism−related genes. (**E**) PPI network of lipid metabolism−related genes arranged by degree in Cytoscape. A large, red circle means that the gene is more important. (**F**) The exhibition of mRNA expression of Ccl2 and Adipoq from GSE146508. * *p* < 0.05, ** *p* < 0.01.

The cutoff for log2FC in plasma was 1.000; 552 genes were downregulated, and 858 genes were upregulated (Figure 4A). The DEGs of plasma are displayed in the heatmap (Figure 4B). Subsequently, we looked for overlapping between lipid metabolism-related genes from the NCBI database and DEGs from the liver and plasma from GSE146508, and four genes (Mest, Adipoq, Ccl2, and Pcsk9) are shown in the Venn diagram (Figure 4C). These intersecting DEGs indicate the close relationship between PM_2.5_ and lipid metabolism.

In addition, the regulation of the lipid metabolic process, the positive regulation of the lipid metabolic process, the neutral lipid metabolic process, and lipid localization pathways are enriched in the GO analysis (*p* value < 0.05, respectively)

The PPI network of 48 overlapped DEGs in liver and lipid metabolism-related genes was illustrated using STRING (Figure 4D) and rearranged in Cytoscape (Figure 4E). Adipoq and Ccl2 are among the top 13 hub genes, and the Adipoq and Ccl2 mRNA expression levels were decreased in the RNA-seq data (Figure 4F). Hub genes Adipoq and Ccl2 are thought to play essential roles in PM_2.5_-related hyperlipidemia. 

### 3.3. Immune Infiltration Analysis

**Figure 5:**
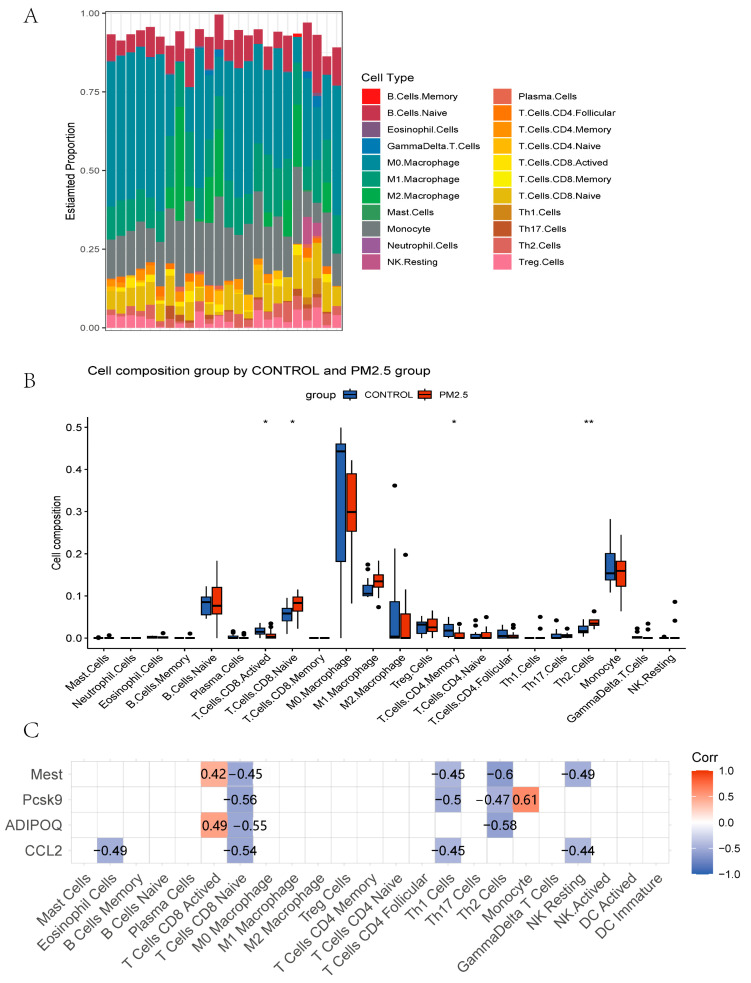
(**A**) Histogram of immune cell composition in model mice liver of GSE146508. (**B**) Box plots of immune cells in PM_2.5_-exposed mice liver and controls from GSE146508. (**C**) Correlation between hub genes CCL2, ADIPOQ, PCSK9, MEST, and immune cells. * *p* < 0.05, ** *p* < 0.01.

**Figure 6:**
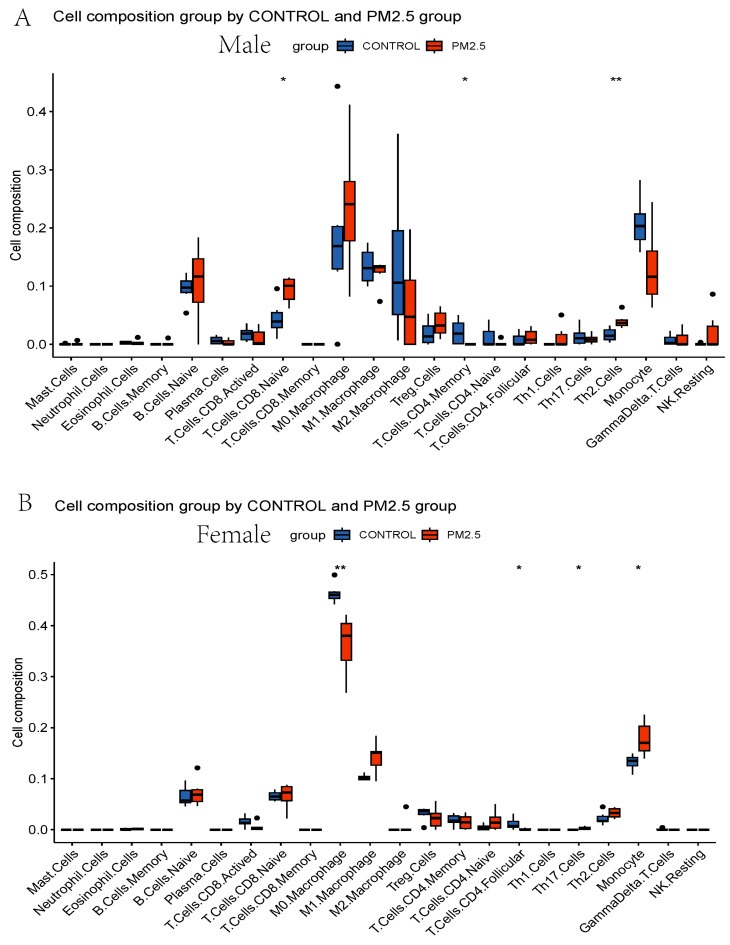
(**A**) Box plots of immune cells in PM_2.5_-exposed male mice liver and controls from GSE146508. (**B**) Box plots of immune cells in PM_2.5_-exposed female mice liver and controls from GSE146508. * *p* < 0.05, ** *p* < 0.01.

Based on the enrichment analysis results, we used the CIBERSORT algorithm to evaluate the immune cell distribution in model mice livers (Figure 5A) and the associations between PM_2.5_ exposure and immune infiltration (Figure 5B). PM_2.5_ exposure is associated with upregulated naïve CD8+ T cells, Th2 cells, and monocytes and downregulated activated CD8+ T cells and memory CD4+ T cells (Figure 5B). Moreover, to understand the immune infiltration difference in sex, the RNAseq data were divided into male and female groups. In male mice, naïve CD8+ T cells and Th2 cells were upregulated, and memory CD4+ T cells were downregulated remarkably in the PM_2.5_ group (Figure 6A). In female mice, monocytes and Th17 cells were increased, and M0 macrophages and follicular CD4+ T cells were decreased significantly in the PM_2.5_ group (Figure 6B).

The four hub genes were significantly associated with different immune cells, indicating the relationship between lipid metabolism-related secretory factors and the PM2.5-affected immune environment (Figure 5C). Adipoq is positively related to activated CD8+ T cells and negatively related to naïve CD8+ T cells and Th2 cells. Ccl2 is negatively related to eosinophils, naïve CD8+ T cells, Th1 cells, and NK resting cells. PCSK9 is highly positively correlated to monocytes, but has a negative relationship with naïve CD8+ T cells, Th1 cells, and Th2 cells. Mest positively correlated with activated CD8+ T cells but is negatively related to naïve CD8+ T cells, Th1 cells, Th2 cells, and NK resting cells.

### 3.4. Lipid and Lipoprotein Profiles in Model Mice Exposed to Intranasal Instillation-Induced Air Pollution

**Figure 7:**
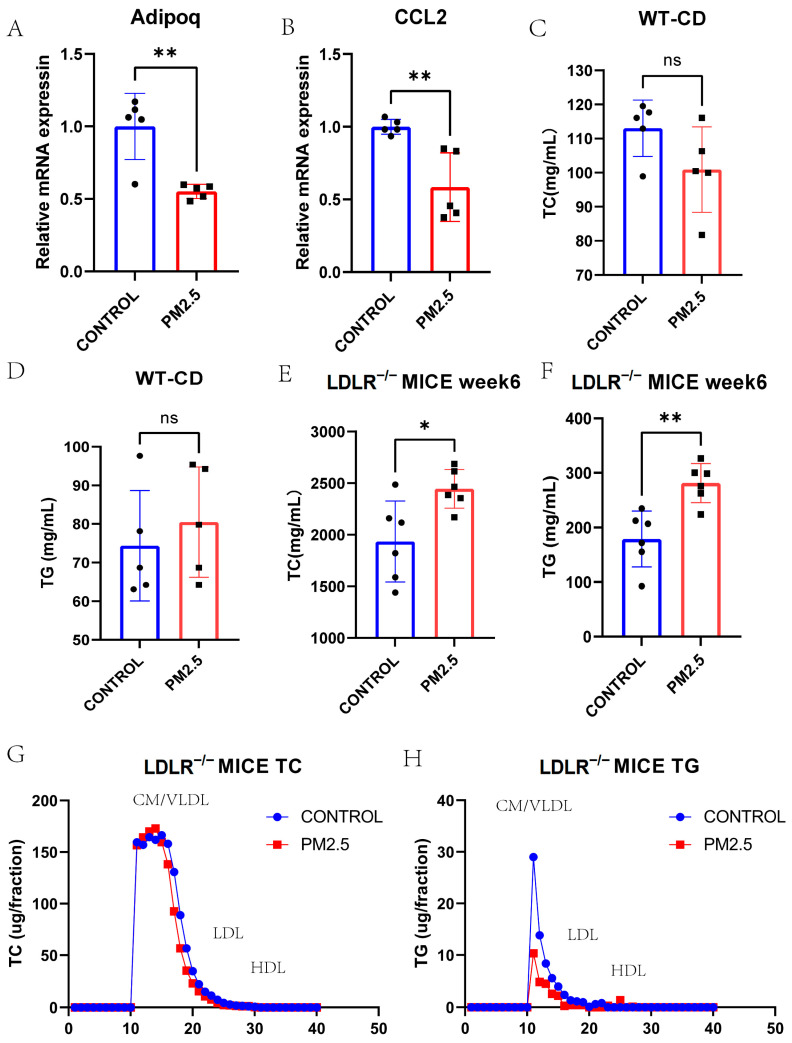
(**A**) QPCR result of Adipoq in PM_2.5_-intranasal-instilled mice liver and controls (*p* = 0.003). (**B**) QPCR result of Ccl2 in PM_2.5_-intranasal-instilled mice liver and controls (*p* = 0.005). (**C**–**F**) Fasting plasma TG and TC levels of PM_2.5_ intranasal instilled WT chow-diet-fed or LDLR^−/−^ HFD-fed mice and controls (*p* = 0.517, 0.109, 0.003, and 0.017, respectively). (**G**,**H**) The distribution of TC and TG in pooled plasma samples of PM_2.5_ intranasal instilled LDLR^−/−^ HFD-fed mice and controls. TC, total cholesterol; TG, triglyceride; WT, wild type; HFD, high fat diet. * *p* < 0.05, ** *p* < 0.01.

Ten 8-week-old male C57BL/6J wild-type (WT) mice and twelve 8-week-old male LDLR^−/−^ mice were all used to construct an air pollution model. Intranasal instillation was performed once every 3 days for 10 times after 2 weeks of chow diet or Paigen diet (PD) feeding. All the animals were sacrificed on the day after the last instillation. First, we tested the mRNA expression levels of Adipoq and Ccl2 using qPCR in the livers of WT model mice exposed to intranasal instillation-induced air pollution or control (Figure 7A,B), which were all decreased. As we can see, the PM_2.5_ and control groups of the WT mice did not have any difference in terms of TC or TG after intranasal instillation (Figure 7C,D). However, the PD-fed LDLR^−/−^ mice after PM_2.5_ intranasal instillation had higher TC and TG levels than the control group (Figure 7E,F, *p* < 0.05).

The FPLC showed a higher CM/VLDL cholesterol level in the PM_2.5_ LDLR^−/−^ mice group. However, the CM/VLDL level of TG in the PM_2.5_ group seems lower than that of the control group (Figure 7G,H).

## 4. Discussion

The effect of PM_2.5_ on hyperlipidemia is inconsistent, but our study confirmed the causation between PM_2.5_ and hyperlipidemia/the TG level from the perspective of genetics. Although the results of previous epidemiological studies showed a contradictory effect of PM_2.5_ on the TG level, we demonstrated that it has a positive effect. MR analysis could avoid inverse causation and potential confounders and evaluate the causality between PM_2.5_ and hyperlipidemia with less susceptibility.

The gene Clcn1 was screened via Mendelian randomization, and its related genes were confirmed in mice transcriptome data. Clcn1 is a member of the Clcn family of voltage-gated chloride ion channels. The Clcn1 channel plays a role in the regulation of muscle excitability and repolarization [43]. These results suggest that muscle contraction-related pathways may be involved in mediating the effect of PM_2.5_ on hyperlipidemia. The function of hub genes Tnnt2 and Myh7 have all been discussed in regard to cardiomyopathy and skeletal muscle myopathy [44,45]. The findings may hint at crosstalk between the liver and other organs, such as the heart and skeletal muscle.

Abnormal lipid metabolism is closely related to the formation of atherosclerosis. In particular, we focused on lipid metabolism-related genes in the liver and plasma transcriptome data from a PM_2.5_ model mice to search for liver-produced secretory factors and speculate on their role in lipid metabolism and atherosclerosis progression. Ccl2 regulates the migration and infiltration of a wide range of immune cells, including monocytes, macrophages, memory T lymphocytes, and natural killer (NK) cells [46,47]. The Ccl2-induced migration of monocytes to the vessel wall is an essential activity contributing to the development of atherosclerosis. During this process [48], Adipoq, also known as adiponectin, is an adipocytokine produced by adipocytes, skeletal, cardiac myocytes, and endothelial cells [49]. Many epidemiological studies suggest that adiponectin deficiency is associated with coronary artery disease [50]. It has been proven that adiponectin prevents endothelial apoptosis through the AMPK-mediated pathway [51] and suppresses the proliferation and migration of vascular smooth muscle cells [52]. Adiponectin is effective in alleviating alcoholic and nonalcoholic fatty liver diseases, including hepatomegaly, steatosis, and elevated levels of serum alanine aminotransferase [53]. Mest has been proven to enlarge adipocytes and could be a marker of the size of adipocytes [54]. Pcsk9 is secreted into the plasma by the liver, binding low-density lipoprotein (LDL) receptors at the surface of hepatocytes, thereby preventing its recycling and enhancing its degradation, resulting in reduced LDL-cholesterol clearance [55]. PCSK9 inhibitors have been produced and recommended in the guidelines for the management of dyslipidemia [56]. The four hub genes related to lipid metabolism are closely associated with atherosclerosis and metabolic syndrome. They play a crucial role in the effect of PM_2.5_ on hyperlipidemia.

At the same time, the CIBERSORT deconvolution algorithm was used to analyze immune cell infiltration in the livers of PM_2.5_ model mice. We could see a significant increase in the number of CD8+ T cells and CD4+ T cells. To further study the sex-related effect of PM_2.5_ on dyslipidemia, the mice were divided into male and female groups. Naive CD8+ T cells and Th2 cells were upregulated, and memory CD4+ T cells were down-regulated in the males. The number of monocytes and Th17 cells was increased, and that of M0 macrophages and follicular CD4+ T cells was decreased in the female mice. This suggests that the difference in immune cell infiltration influenced by PM_2.5_ is closely related to sex [57]. The increased monocyte and decreased M0 macrophage numbers in the female mice indicated that monocytes were recruited after exposure to PM_2.5_, and the phenotypic transformation of macrophages happened in the PM_2.5_-exposed female mice. However, CD8+ T cells and CD4+ T cells are a few resident cells recruited rapidly during a liver infection or injury [58]. The number of naive CD8+ T cells and Th2 cells (a subtype of CD4+ T cells) was increased in the PM_2.5_-exposed male mice, and that of Th17 cells (a subtype of CD4+ T cells) was increased in the PM_2.5_-exposed female mice, which means the immune reaction was similar to that which would occur during a liver infection. This reaction may lead to the formation of tertiary immune structures, which are also known as intrahepatic myeloid cell aggregates for T cell population expansion (iMATEs) [59]. Meanwhile, we found that the potential secretory factors affecting lipid metabolism and the atherosclerosis process also have positive or reverse relationships with different types of immune cells.

Finally, we established intranasal instillation models of WT mice and LDLR^−/−^ mice to measure the difference in TC and TG levels between the saline and PM_2.5_ groups. At the same time, a difference in lipoprotein distribution was observed via the FPLC method. The increase in CM/VLDL cholesterol is supposed to be the cause of PM_2.5_-induced atherosclerosis.

Advantages and innovations: 1. The Mendelian randomization method was used for the first time to explore the relationship between PM_2.5_ and hyperlipidemia in this population; transcriptome data were used as validation. 2. Focusing on the influence of PM_2.5_ on lipid metabolism, we searched for hub genes, enriched the pathways related to lipid metabolism, and performed qPCR verification. 3. We established an animal model of air pollution in which WT mice simulating healthy people and LDLR^−/−^ mice simulating FH patients were utilized. We focused on the blood lipids of WT mice fed a chow diet and LDLR^−/−^ mice under PD feeding conditions and discussed the differences in the blood lipids and the distribution of lipoproteins in the LDLR^−/−^ mice in the air pollution model for the first time.

Limitations: 1. Although the gene Clcn1 selected via MR was certificated in the enrichment pathways from mice liver RNA-seq data, it seemed to have a minimal association with hyperlipidemia. Therefore, we focused on the lipid metabolism pathways which were less significant in the enrichment analysis. 2. The plasma lipid levels of wild-type mice fed a High-Fat Diet (HFD) and LDLR^−/−^ mice fed a chow diet could be further tested to study the effect of different diets during PM_2.5_ exposure. According to the different immune cell infiltration in male and female mice livers, female mice could also be utilized to identify the sex-related effect of PM_2.5_ on dyslipidemia. RNA transcriptome and single-cell RNA sequencing can be further performed on the livers of LDLR^−/−^ mice to analyze the differences in PM_2.5_ and control groups in terms of lipid metabolism after the amplification of HFD. 3. Dyslipidemia is closely related to atherosclerosis, so the effect and mechanism of PM_2.5_ on atherosclerosis can be further discussed.

## 5. Conclusions

Our study revealed the causality between PM_2.5_ and hyperlipidemia using genome-wide Mendelian randomization. Four possible proteins secreted from the liver to plasma were excavated from RNAseq data, which might accelerate atherosclerosis. The immune cell infiltration of PM_2.5_ exposed mice liver was explored, and a sex-related effect was found. The lipid profile and lipoprotein distribution of LDLR^−/−^ PM_2.5_ model mice supplement the research on the dyslipidemia of FH patients affected by PM_2.5_. The results from GWAS, RNAseq, and the animal model all conclude that PM_2.5_ could aggravate dyslipidemia and maybe accelerate atherosclerosis.

## Figures and Tables

**Table 1:** Mendelian randomization estimates of the causal relationships between PM_2.5_ and hyperlipidemia.

Exposure	Outcome	Method	snp	OR	SE	*p* Value
ukb-b-10817	ukb-b-17462	MR Egger	60	1.036497	0.016453	0.033427
ukb-b-10817	ukb-b-17462	Weighted median	60	1.003108	0.003327	0.351026
ukb-b-10817	ukb-b-17462	Inverse variance weighted	60	1.006341	0.002738	0.020954
ukb-b-10817	ukb-b-17462	Simple mode	60	1.000313	0.007391	0.966405
ukb-b-10817	ukb-b-17462	Weighted mode	60	1.001635	0.006905	0.813814

**Table 2:** Mendelian randomization estimates of the causal relationships between PM_2.5_ and TG level.

Exposure	Outcome	Method	snp	OR	SE	*p* Value
ukb-b-10817	met-d-Total_TG	MR Egger	102	1.036998	0.116365	0.75553
ukb-b-10817	met-d-Total_TG	Weighted median	102	1.075608	0.05714	0.202108
ukb-b-10817	met-d-Total_TG	Inverse variance weighted	102	1.100426	0.045397	0.035029
ukb-b-10817	met-d-Total_TG	Simple mode	102	1.125343	0.177939	0.508431
ukb-b-10817	met-d-Total_TG	Weighted mode	102	1.125343	0.1713	0.492174

## Data Availability

SNP data of ukb-b-10817, ukb-b-17462, and met-d-Total_TG are available at https://gwas.mrcieu.ac.uk/ accessed on 25 July 2023. We searched the RNA-seq data of GSE146508 on the Gene Expression Omnibus (GEO, http://www.ncbi.nlm.nih.gov/geo, accessed on 21 August 2023) database.

## Supplementary Materials

The following supporting information can be downloaded at: https://www.mdpi.com/article/10.3390/toxics11100823/s1, Table S1, Studies about air pollution and dyslipidemia; Table S2, Mice primers of qPCR.
